# Assessing the Accuracy and Reliability of AI-Generated Medical Responses: An Evaluation of the Chat-GPT Model

**DOI:** 10.21203/rs.3.rs-2566942/v1

**Published:** 2023-02-28

**Authors:** Douglas Johnson, Rachel Goodman, J Patrinely, Cosby Stone, Eli Zimmerman, Rebecca Donald, Sam Chang, Sean Berkowitz, Avni Finn, Eiman Jahangir, Elizabeth Scoville, Tyler Reese, Debra Friedman, Julie Bastarache, Yuri van der Heijden, Jordan Wright, Nicholas Carter, Matthew Alexander, Jennifer Choe, Cody Chastain, John Zic, Sara Horst, Isik Turker, Rajiv Agarwal, Evan Osmundson, Kamran Idrees, Colleen Kieman, Chandrasekhar Padmanabhan, Christina Bailey, Cameron Schlegel, Lola Chambless, Mike Gibson, Travis Osterman, Lee Wheless

**Affiliations:** Vanderbilt University Medical Center; Vanderbilt University School of Medicine; Vanderbilt University Medical Center; Vanderbilt University Medical Center, Nashville, Tennessee; Vanderbilt University Medical Center; Vanderbilt University Medical Center; Vanderbilt University Medical Center; Vanderbilt University Medical Center; Vanderbilt University Medical Center; Vanderbilt University Medical Center; Vanderbilt University Medical Center; Vanderbilt University Medical Center; Vanderbilt University Medical Center; Vanderbilt University Medical Center; Vanderbilt University Medical Center; Vanderbilt University Medical Center; Vanderbilt University Medical Center; Vanderbilt University Medical Center; Vanderbilt University Medical Center; Vanderbilt University Medical Center; Vanderbilt University Medical Center; Vanderbilt University Medical Center; Vanderbilt University Medical Center; Vanderbilt University Medical Center; Vanderbilt University Medical Center; Vanderbilt University Medical Center; Vanderbilt University Medical Center; Vanderbilt University Medical Center; Vanderbilt University Medical Center; Vanderbilt University Medical Center; Vanderbilt University; Vanderbilt University Medical Center; Vanderbilt University Medical Center; Vanderbilt University Medical Center

**Keywords:** artificial intelligence, large language model, natural language processing, ChatGPT, knowledge dissemination, clinical decision making, medical education, deep learning

## Abstract

**Background:**

Natural language processing models such as ChatGPT can generate text-based content and are poised to become a major information source in medicine and beyond. The accuracy and completeness of ChatGPT for medical queries is not known.

**Methods:**

Thirty-three physicians across 17 specialties generated 284 medical questions that they subjectively classified as easy, medium, or hard with either binary (yes/no) or descriptive answers. The physicians then graded ChatGPT-generated answers to these questions for accuracy (6-point Likert scale; range 1 – completely incorrect to 6 – completely correct) and completeness (3-point Likert scale; range 1 – incomplete to 3 - complete plus additional context). Scores were summarized with descriptive statistics and compared using Mann-Whitney U or Kruskal-Wallis testing.

**Results:**

Across all questions (n=284), median accuracy score was 5.5 (between almost completely and completely correct) with mean score of 4.8 (between mostly and almost completely correct). Median completeness score was 3 (complete and comprehensive) with mean score of 2.5. For questions rated easy, medium, and hard, median accuracy scores were 6, 5.5, and 5 (mean 5.0, 4.7, and 4.6; p=0.05). Accuracy scores for binary and descriptive questions were similar (median 6 vs. 5; mean 4.9 vs. 4.7; p=0.07). Of 36 questions with scores of 1-2, 34 were re-queried/re-graded 8-17 days later with substantial improvement (median 2 vs. 4; p<0.01).

**Conclusions:**

ChatGPT generated largely accurate information to diverse medical queries as judged by academic physician specialists although with important limitations. Further research and model development are needed to correct inaccuracies and for validation.

## Introduction

The integration of natural language processing (NLP) models in healthcare has the potential to radically enhance the accessibility of medical information for health professionals and patients. Large language models (LLMs) are NLP tools that can understand and generate human-like text. Compared to traditional supervised deep learning models, large language models can learn from data more efficiently with a two-stage training process, starting with self-supervised learning on vast amounts of unannotated data, then fine-tuned on smaller, task-specific, annotated datasets so they can perform on end-user-specified tasks (1)..

One such AI-powered tool that has gained recent widespread popularity is Chat-Generative Pre-Trained Transformer (ChatGPT), a conversational chatbot based on Generative-Pre-Trained Transformer-3.5 (GPT-3.5), an LLM with over 175 billion parameters (2). ChatGPT’s training data encompasses a broad range of internet sources, including books, articles, and websites. Fine-tuning for conversational tasks using reinforcement learning from human feedback (3), allows ChatGPT to incorporate the complexity of users’ intentions, thus enabling it to proficiently respond to various end-user tasks, potentially including medical queries.

With the increasing amount of medical data and complexity of clinical decision-making, NLP tools could theoretically assist physicians in making timely, informed decisions and improve the overall quality and efficiency of healthcare. ChatGPT performed at or near the passing threshold for United States Medical Licensing Exam (USMLE) without any specialized training, suggesting its potential for medical education and clinical decision support (4, 5). Further, technology advancements have led to the democratization of knowledge, where patients no longer solely rely on healthcare professionals for medical information. Instead, they are increasingly turning to search engines, and now AI chatbots, as convenient and accessible sources of medical information. ChatGPT and other recently released chat-bots engage in conversational interactions and provide authoritative-sounding responses to complicated medical queries. However, despite its potential, ChatGPT often produces seemingly credible but incorrect outputs, thus warranting caution when considering its applications in medical practice and research (6–11). The reliability and accuracy of these engines has not been assessed, particularly in the context of open-ended medical questions that physicians and patients are likely to ask.

This study aims to evaluate the accuracy and comprehensiveness of ChatGPT-generated responses to medical queries developed by physicians. This will provide an early evidence base on the reliability of ChatGPT for providing accurate and complete information. Additionally, this study will highlight limitations of AI-provided medical information.

## Methodology

This study was IRB exempt as no patient-level data were used. A dataset of questions was generated by 33 physicians across 17 medical, surgical, and pediatric specialties (**Table S1**). A total of 59 physicians were originally invited to participate (56% response rate); all respondents were faculty (N=31) or recent graduates from residency or fellowship programs (N=2) at a single academic medical center (Vanderbilt University Medical Center). At least 1 physician from each major specialty was invited to participate. These physicians were instructed to provide questions with clear and uncontroversial answers from available medical guidelines, and unchanged from the beginning of 2021 (accounting for the cut-off of the training set for ChatGPT). Each physician was asked to produce six questions, three of which had binary yes/no or right/wrong answers and were rated as easy, medium, and hard by subjective rating by the physicians who provided the questions. The other three questions had answers that were either descriptive in nature or produced a list of multiple correct answers, with the same three complexity ratings. To minimize bias, the physicians were asked not to screen the questions themselves in ChatGPT. To assess physician agreement and to generate additional data, the senior authors (LEW and DBJ) provided and rated a second dataset of 44 melanoma and immunotherapy-specific questions. Finally, since most participating physicians were specialists, a third dataset of 60 questions encompassing 10 common medical conditions (see **Table S2**) were also produced and rated by the senior authors. Six questions were generated for each common medical condition with the same question classification (binary vs. descriptive, and easy vs. medium vs. hard difficulty level). All questions were subjectively chosen as representative of each physician’s subject matter expertise.

To ensure consistency, all questions were entered into the ChatGPT engine by one investigator (RG), who prompted the chatbot to be specific and incorporate any medical guidelines into the answer if appropriate (with the phrase “Be specific and incorporate any applicable medical guidelines”). The AI-generated answers were then provided to the physicians who created the questions. Based on their medical expertise in their corresponding field, the physicians rated the accuracy of answers according to two predefined scales of accuracy and completeness.

The accuracy scale was a six-point Likert scale (1 – completely incorrect, 2 – more incorrect than correct, 3 – Approximately equal correct and incorrect, 4 – more correct than incorrect, 5 – nearly all correct, 6 – correct), and the completeness scale was a three-point Likert scale (1 – incomplete, addresses some aspects of the question, but significant parts are missing or incomplete, 2 – adequate, addresses all aspects of the question and provides the minimum amount of information required to be considered complete, 3 – comprehensive, addresses all aspects of the question and provides additional information or context beyond what was expected). Answers that were completely incorrect on the accuracy scale (score of 1) were not graded on comprehensiveness.

To assess the reproducibility of the results and evaluate the effect of time on answer accuracy, an internal validation process was conducted in which ChatGPT was re-queried with the same questions that generated answers originally graded as inaccurate (less than 3 on the accuracy scale). The re-scored answers were generated by ChatGPT 8 to 17 days after the initial answers, and physicians re-scored the updated AI answers.

Score results were listed descriptively (median, mean, intra-quartile range, standard deviation), and were compared between groups using Mann-Whitney U or Kruskal Wallis testing. Re-graded questions were compared using the Wilcoxon signed rank test. In the melanoma/immunotherapy, and the common conditions dataset, intra-rater agreement was graded using the kappa statistic across all scores (1-6 for accuracy and 1-3 for completeness) and in a condensed, exploratory fashion to assess for general agreement (agreement for accuracy scores 1-2 vs. 3-4 vs. 5-6).

## Results

### Multispecialty Analysis

**Figure 1:**
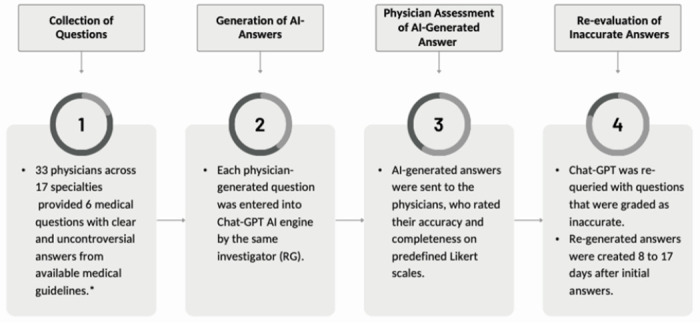
Methodology *DBJ and LEW scored two separate datasets of melanoma and immunotherapy and common conditions questions.

ChatGPT-generated answers were initially evaluated based on 180 questions provided by 33 physicians from 17 specialties. As noted, each physician provided three descriptive and three binary questions at different difficulty levels (easy, medium, and hard) except for one author who provided two descriptive question sets (Figure 1). An example of an easy-level difficulty descriptive question was, “What are the first-line treatments for Stage IA mycosis fungoides?” An example of a medium-level difficulty descriptive question was, “Which patients with well-differentiated thyroid cancer should receive postoperative radioactive iodine ablation?” An example of a hard-level difficulty binary question was, “Can we start angiotensin receptor-neprilysin inhibitors immediately after discontinuing an angiotensin-converting enzyme inhibitor?” For additional example questions and answers, see Table 1.

Among 180 ChatGPT-generated answers, the median accuracy score was 5 (mean 4.4, SD 1.7), and the median completeness score was 3 (mean 2.4, SD 0.7) (Table 2). 39.4% (n= 71) were scored at the highest level of accuracy (accuracy score of 6) and 18.3% (n= 33) were scored as nearly all correct (accuracy score of 5). Conversely, 8.3% (n=15) of answers were scored as completely incorrect (accuracy score of 1). Inaccurate answers, receiving accuracy scores of 2 or lower (n=36), were most commonly in response to physician-rated hard questions with either binary answers (n=8, 22%) or descriptive answers (n=7, 19%), but were distributed across all categories. Additionally, the completeness of answers was evaluated, with 53.3% (n = 96) scored as comprehensive, 26.1 % (n = 47) as adequate, and 12.2% (n = 22) as incomplete. Fifteen (8.3%) answers did not receive completeness ratings due an accuracy score of 1 (completely incorrect). Accuracy and completeness were modestly correlated (Spearman’s r = 0.4, 95% CI 0.3 to 0.5, p < 0.01, alpha = 0.05) across all questions.

#### Question Type and Difficulty Level

Among both descriptive and binary questions, the median accuracy scores for easy, medium, and hard answers were 5 (mean 4.6, SD 1.7, IQR 3), 5 (mean 4.3, SD 1.7, IQR 3), and 5 (mean 4.2, SD 1.8, IQR 3.8), respectively, similar between groups (Kruskal Wallis p = 0.4). The median completeness scores for all answers were 3 (mean 2.6, SD 0.7, IQR 1), 3 (mean 2.4, SD 0.7, IQR 1), and 2.5 (mean 2.4, 0.7, IQR 1) respectively, with no differences in completeness based on difficulty (Kruskal Wallis p = 0.3).

**Figure 2:**
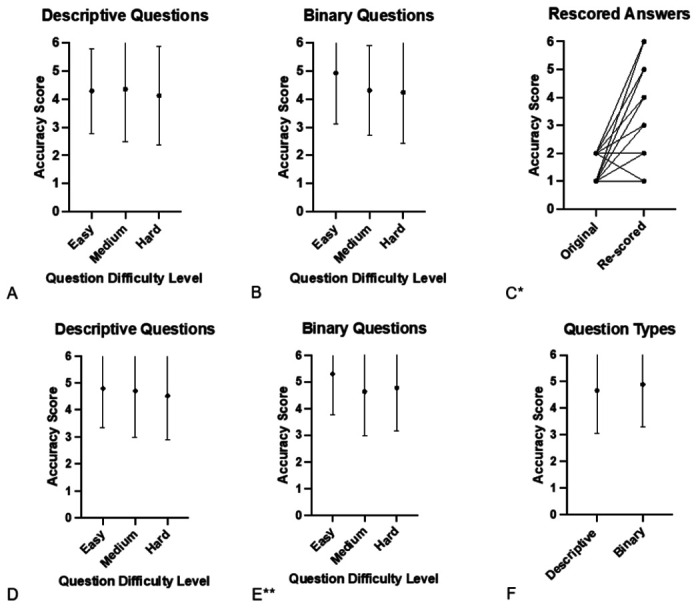
Accuracy of Chat-GPT-Generated Answers Accuracy of AI answers from multispecialty questions (A-C) or all questions (multispecialty, melanoma and immunotherapy, and common medical conditions; D-F). *p < 0.01, ** p = 0.03

Both descriptive and binary questions were analyzed to assess ChatGPT’s performance on these distinct categories. The median accuracy score of descriptive questions (n=93) was 5 (mean 4.3, SD 1.7, IQR 3) and the mean accuracy score of binary questions (n=87) was also 5 (mean 4.5, SD 1.7, IQR 3), similar between groups (Mann Whitney U p=0.3) (Table 2). Among descriptive questions, the median accuracy scores for easy, medium, and hard questions were 5 (mean 4.9, SD 1.5, IQR 3), 5 (mean 4.4, SD 1.9, IQR 3), and 5 (mean 4.1, SD 1.8, IQR 3), respectively (Kruskal Wallis p = 0.7) (Table 2, Figure 2A).

Among binary questions, the median accuracy scores for easy, medium, and hard answers were 6 (mean 4.9, SD 1.8, IQR 1), 4 (mean 4.3, SD 1.6, IQR 3), and 5 (mean 4.2, SD 1.8, IQR 4), respectively, without statistically significant difference (Kruskal Wallis p = 0.1) (Table 2, Figure 2B). Overall, the results suggested no major differences in the accuracy and completeness of ChatGPT-generated answers for descriptive or binary questions across levels of difficulty.

### Internal Validation: Re-scored Analysis

Of 36 inaccurate answers that received a score of 2 or lower on the accuracy scale, 34 were re-scored by physicians to evaluate the reproducibility of answers over time (Table 3). Notably, scores generally improved with 26 questions improving, 7 remaining the same, and 1 decreasing in accuracy. Median accuracy scores for the original questions was 2 (mean 1.6, SD 0.5, IQR 1) compared with median 4 (mean 3.9, SD 1.8, IQR 3.3) for re-scored answers(Wilcoxon signed rank p<0.01) (**Table S3**, Figure 2C). The re-scored answers were generated from ChatGPT 8 to 17 days after the initial answers were generated.

### Melanoma and Immunotherapy Analysis

To further assess performance and judge intra-rater variability, two physicians (DBJ and LEW) independently assessed additional questions on melanoma diagnosis and treatment as well as cancer immunotherapy use from existing guidelines before 2021. Among 44 AI-generated answers, the median accuracy score was 6 (mean 5.2, SD 1.3, IQR 1), and the median completeness score was 3 (mean 2.6, SD 0.8, IQR 0.5) (Table 2). The median accuracy scores of descriptive and binary questions were 6 (mean 5.1, SD 1.5, IQR 1) and 6 (mean 5.4, SD 1.2, IQR 1), respectively (Mann-Whitney U p = 0.7). Among both descriptive and binary questions, the median accuracy scores for easy, medium, and hard answers were 6 (median 5.9, SD 0.3, IQR 0), 5.5 (mean 4.8, SD 1.7, IQR 2.1), and 5.8 (mean 5.3, SD 1.1, IQR 1), respectively, with a significant trend (Kruskal Wallis p = 0.046). There was fair interrater agreement (kappa = 0.3, SE 0.1, 95% CI 0.1-0.6) for accuracy and moderate agreement (kappa = 0.5, SE 0.2, 95% CI 0.2 to 0.8) for completeness (**Table S4**). When 6 accuracy categories were condensed into 3 subgroups (1– 2, 3–4, 5–6), inter-rater agreement for accuracy was moderate (kappa = 0.5, SE 0.2, 95% CI 0.2-0.8).

### Common Conditions Analysis

To assess performance further in general questions widely pertinent across practitioners, the same two physicians (LEW and DBJ) generated and graded questions related to ten common medical conditions (**Table S2**). Among 60 AI-generated answers, the median accuracy score was 6 (mean 5.7, SD 0.7, IQR 0.3), and the median completeness score was 3 (mean 2.8, SD 0.5, IQR 0). The median accuracy score of descriptive questions was 6 (mean 5.6, SD 0.6, IQR 0.5) and the median accuracy score of binary questions was 6 (mean 5.8, 0.8, IQR 0.1) (Mann-Whitney U p=0.1) Among both descriptive and binary questions, the median accuracy scores for easy, medium, and hard answers were 6 (mean 5.9, SD 0.4, IQR 0), 6 (mean 5.6, SD 1.0, IQR 0.5), and 6 (mean 5.6, SD 0.1, IQR 0.5), respectively (Kruskal Wallis p = 0.07). There was slight interrater agreement (kappa = 0.4, SE 0.1, 95% CI 0.1-0.6) for accuracy and moderate agreement (kappa = 0.2, SE 0.1, 95% CI 0.01 to 0.4) for completeness (**Table S5**). When 6 accuracy categories were grouped into 3 subgroups (1-2, 3-4, 5-6), inter-rater agreement for accuracy was moderate (kappa = 0.5, SE 0.2, 95% CI 0.03-0.9).

### Total Analysis

Among all AI-generated answers (n=284) from all three datasets (not including re-graded answers), the median accuracy score was 5.5 (median 4.8, SD 1.6, IQR 2), and the median completeness was 3 (mean 2.5, SD 0.7, IQR 1) (Table 2). The median accuracy of all descriptive questions was 5 (mean 4.7, SD 1.6, IQR 2.6), and the median accuracy of binary questions was 6 (mean 4.9, SD 1.6, IQR 2) (Mann Whitney U p=0.07). Among the descriptive questions, the median accuracy scores for easy, medium, and hard questions were 5.25 (mean 4.8, SD 1.5, IQR 3), 5.5 (mean 4.7, SD 1.7, IQR 2.8), and 5 (mean 4.5, 1.6, IQR 2.4), respectively (Kruskal-Wallis p = 0.4) (Figure 2D). Among binary questions, median accuracy scores for easy, medium, and hard questions were 6 (mean 5.3, SD 1.5, IQR 1), 5.5 (mean 4.6, SD 1.6, IQR 2.6), and 5.5 (mean 4.8, SD 1.6, IQR 2), respectively, which resulted in a significant difference among groups (Kruskal-Wallis p = 0.03) (Figure 2E).

## Discussion

This study indicates that three months into its existence, ChatGPT has promise for providing accurate and comprehensive medical information. However, it remains well short of completely reliable. The multispecialty analyses of 180 questions provided by 33 physicians across 17 specialties revealed 57.8% (n=104) of AI answers were rated as “nearly all correct” or “correct” (mean accuracy score 4.4, median 5). Most (53.5%) answers were comprehensive (mean completeness score 2.4, median 3), indicating complete answers with additional information or context. When analyzing the data, the median accuracy scores were generally higher than mean scores, which was reflective of multiple instances where the chatbot was spectacularly and surprisingly wrong. Thus, any use of the current version of ChatGPT for medical knowledge dissemination must consider its capacity to come to a totally mistaken conclusion, which is delivered in an authoritative and convincing manner.

Overall, accuracy was fairly high across question types and difficulty. More difficult questions seemed to have perhaps slightly less accurate scores (mean 4.2) than easier questions (mean 4.6), suggesting a potential limitation of the model in handling complex medical queries, but this did not reach statistical significance. Overall though, the results for type of question (descriptive or binary) or difficulty level were broadly similar, implying ChatGPT could have promise for open-ended question types with varying levels of difficulty, providing potential broad applicability.

An internal validation process demonstrated ChatGPT’s potential to improve significantly over a short period of time. The re-scored answers were generated from ChatGPT only 8 to 17 days after the initial responses were generated. Compared with the median accuracy score of 2 (median 2, SD 0.5, IQR 1) for the original low-quality answers, median accuracy score improved to 4 (p < 0.01) (Table 2). This improvement could be attributed to the continuous update and refinement of the model’s algorithms and parameters and the impact of repetitive user feedback through reinforcement learning.

Analysis results from the three distinct question sets (multispecialty, melanoma and immunotherapy, and common conditions) revealed fairly similar and high ratings. However, the common conditions dataset had somewhat higher accuracy scores, suggesting that more common conditions may have more training data available, leading to improved scores. However, this dataset was also scored later, thus potentially reflecting model improvement over days to weeks.

Despite these promising results, the scope of our conclusions is limited due to the modest sample size of 33 physicians from a single academic medical center, and the dataset of 284 questions, which may not be representative of all medical specialties and the many questions that can be posed within them. Further, selection bias of a cohort of physicians limited to those in academic practice, as well as respondent bias were present. Other limitations include subjective choice of questions and the absence of a validation mechanism to verify the accuracy of the information provided. The study also relied on physicians’ subjective, self-reported ratings, which may introduce bias; similar judgements may also vary by physician (as the difference between more correct than incorrect vs. nearly all correct (4 vs. 5) may be small). Questions chosen by physicians were those with clear and uncontroversial answers and included instructions to incorporate current guidelines, and are thus likely not representative of queries that patients and the general public would make. In particular, many patients may not have the explicit knowledge to incorporate, for example, cancer staging, prior therapies, sites of metastases, all of which may impact answers. The analyses were limited to a specific AI model, ChatGPT, and may not apply to other AI models, particularly those with medical-specific training.

This study provide an early evidence base to demonstrate the potential of AI-based systems to provide answers to real-world, non-multiple choice clinical questions. ChatGPT or similar tools, with further validation, could serve as a valuable resource for rapid medical information retrieval in a fast-paced clinical setting to enhance healthcare efficiency and complex decision making. Healthcare professionals should also consider how patients may use these tools, and how ChatGPT is programmed to provide appropriate recommendations and referrals to qualified health professionals. Medical education should include training on the potential benefits, limitations and risks associated with AI-powered tools, so that both healthcare professionals and patients can make informed decisions about when and how to use them. At the same time, relying solely on the current, publicly available version of ChatGPT as a sole source for medical information is not advisable. If trained by reliable experts and on a larger dataset of vetted medical information (medical literature, pharmacology databases, electronic medical records, etc.), large language models like ChatGPT have the potential to rapidly improve and transform dissemination of medical knowledge. A recently released GPT-style language model trained exclusively on biomedical literature to enhance domain-specific language model training achieved 51% accuracy on various biomedical question answering tasks (12). This highlights the promise of domain-specific language generation models in real-world applications in healthcare.

Further research is needed to validate the reliability of AI-provided medical information with large groups of healthcare professionals and diverse question types. Additionally, studies should assess the evolution of AI-generated medical information over time. Other important considerations include ethical and privacy concerns. Because these AI tools are only as reliable as the data they are trained on, efforts should be made to incorporate reliable medical information sources such as medical literature, pharmacology databases, and real-world evidence to ensure that AI models are well-informed and provide the most up-to-date information. Moreover, models that are trained exclusively on text might miss nuances presented only in figure or tabular form and not discussed specifically in the body of a paper. Finally, future efforts should focus on developing nimble though robust standards and regulations for the safe and effective implementation of AI in healthcare.

## Conclusion

Integrating language models, such as ChatGPT in medical practice reveals early promise, but with many considerations for safe and optimal use. While the AI-generated answers displayed high accuracy and completeness scores across various specialties, question types, and difficulty levels, further development is needed to improve the reliability and robustness of these tools before clinical integration. Medical professionals and patients should be aware of the limitations and actively check AI-generated medical information with trusted sources. This study is a foundational step towards establishing an evidence base for using AI language models in healthcare and highlights the importance of ongoing evaluation and regulation.

## Figures and Tables

**Table 1.** Examples of Chat-GPT Generated Answers to Medical Questions from Physicians

Multispecialty– Descriptive Questions					
Question Difficulty Level	Specialty	Physician-generated questions	AI-generated answers	Accuracy Rating	Completeness Rating
Easy	Ophthalmology	What is the first line of treatment for patients with neovascular age-related macular degeneration?	The first line of treatment for neovascular age-related macular degeneration (AMD) is typically anti-vascular endothelial growth factor (VEGF) therapy, which targets the abnormal blood vessels that form in the retina. This can include intravitreal injections of medications such as ranibizumab, aflibercept, or bevacizumab. These medications are typically administered on a regular schedule, such as every 4-8 weeks, to maintain the effects of the treatment. According to the American Academy of Ophthalmology, treatment should be initiated as soon as possible after diagnosis to prevent vision loss.	6	3
Easy	Infectious Disease	What oral antibiotics may be used for the treatment of methicillin-resistant *Staphylococcus aureus* infections?	Oral antibiotics that may be used for the treatment of methicillin-resistant Staphylococcus aureus (MRSA) infections include: • Doxycycline • Clindamycin • Tetracycline • Minocycline • Linezolid • Daptomycin • Telavancin It is important to note that treatment of MRSA infections should be guided by susceptibility testing and the clinical scenario, and treatment should be initiated in consultation with an infectious disease specialist.	3	1
Medium	Neurological Surgery	What are the indications for surgical resection in a patient with a prolactinoma?	The indications for surgical resection of a prolactinoma, a type of pituitary tumor that secretes the hormone prolactin, typically include: 1. Persistent symptoms despite medical therapy: Patients who have symptoms such as headaches, visual disturbances, or hypogonadism that persist despite treatment with dopamine agonists, the primary medical therapy for prolactinomas, may be considered for surgery. 2. Tumor size: Prolactinomas that are larger than 10 mm in diameter are more likely to cause symptoms and may be more difficult to control with medical therapy alone. These tumors may be considered for surgery. 3. Macroadenomas: Prolactinomas that are classified as macroadenomas, tumors that are larger than 10 mm in diameter, are more likely to cause symptoms and may be more difficult to control with medical therapy alone. These tumors may be considered for surgery. 4. Tumor invasion: Prolactinomas that have invaded surrounding structures, such as the optic chiasm or cavernous sinus, may be considered for surgery to prevent further invasion and preserve neurological function. 5. Pregnancy: If a woman with a prolactinoma is planning to become pregnant, surgery may be considered to control the prolactin level and prevent the tumor from growing during pregnancy. It’s important to note that surgery is not always necessary for prolactinomas and that the decision to proceed with surgery should be made on a case-by-case basis. A multidisciplinary team of endocrinologists and neurosurgeons should be consulted to provide the best treatment plan for the patient.	5	2
Medium	Gastroenterology	What medications are contraindicated for a patient with UC during pregnancy?	During pregnancy, the use of certain medications may be contraindicated due to the potential risks to the mother and/or the developing fetus. Medications that are contraindicated for use during pregnancy in patients with UC include: • Sulfasalazine: It is classified as a category D pregnancy drug, meaning that there is positive evidence of human fetal risk, but the benefits from use in pregnant women may be acceptable despite the risk. • Methotrexate: It is classified as a category X pregnancy drug, meaning that it is contraindicated during pregnancy because it may cause fetal harm • Cyclosporine and Tacrolimus: They are classified as a category C pregnancy drug, meaning that there is a risk to the developing fetus, but the benefits may outweigh the risks in certain cases. • Biologies (such as Infliximab, Adalimumab, Vedolizumab, Ustekinumab, Certolizumab pegol, Golimumab) is generally avoided during pregnancy as their safety in pregnancy is not well established yet. In addition to the medications mentioned above, there are other medications that may be contraindicated for use during pregnancy in patients with UC. These include: . Corticosteroids: such as prednisone, budesonide, hydrocortisone, etc. These medications may be used to manage inflammation and symptoms of UC, but their long-term use during pregnancy is not recommended due to the potential risks to the developing fetus. . Azathioprine and Mercaptopurine: These medications are classified as category D pregnancy drugs and may be used to manage UC during pregnancy, but their use should be carefully weighed against the potential risks to the mother and fetus. . 5-aminosalicylic acid (5-ASA) drugs: such as mesalamine and sulfasalazine, are considered as category B pregnancy drugs, meaning that they are generally considered safe during pregnancy, but their long-term use should be avoided. It’s important to note that these medications may be used during pregnancy but only under the guidance of healthcare provider and after a thorough risk-benefit analysis, as well as considering alternatives such as lifestyle modifications and other medications that may be safer during pregnancy.	1	n/a
Hard	Radiation Oncology	What radiotherapy dose and fractionation schemes are standard of care for first line treatment of T2N2M0 small cell lung cancer?	The standard of care for first-line treatment of T2N2M0 small cell lung cancer (SCLC) is concurrent chemoradiotherapy, which involves the administration of both radiation therapy and chemotherapy at the same time. The recommended radiation dose and fractionation scheme for the treatment of T2N2M0 SCLC is 60-66 Gy in 30-33 fractions over 6-7 weeks. This is based on the results of multiple studies and clinical trials that have shown that this dose and fractionation scheme is effective in controlling the cancer while minimizing the risk of side effects. The concurrent chemotherapy regimen is usually etoposide and cisplatin or carboplatin, which are drugs that have been shown to be effective in treating SCLC. The chemotherapy is usually given once per week during the radiation therapy treatment. It is important to note that the best treatment option for an individual patient will depend on many factors such as the specific characteristics of their tumor, their overall health, and their preferences. It is recommended that patients with T2N2M0 SCLC discuss the full range of treatment options with their oncologist to determine the best course of treatment for them.	4	3
Hard	Trauma Surgery	What are the key principles of managing necrotizing soft tissue infection?	The key principles of managing necrotizing soft tissue infections are: 1. Early recognition and aggressive surgical debridement: The goal is to remove all necrotic tissue as soon as possible to prevent the spread of infection. 2. Broad-spectrum antibiotics: Necrotizing infections are polymicrobial, so the use of broad-spectrum antibiotics is recommended to cover a wide range of potential pathogens. 3. Intensive supportive care: Patients with necrotizing infections are often critically ill and may require intensive care support, including fluid and electrolyte management, wound care, and close monitoring of vital signs. 4. The IDSA (Infectious Diseases Society of America) guidelines recommends early aggressive surgical debridement and broad-spectrum antimicrobial coverage as the primary treatment of necrotizing soft tissue infections. In addition to the key principles already mentioned, there are several other important aspects to consider when managing necrotizing soft tissue infections: 1. Prompt surgical consultation: Patients with suspected necrotizing infections should be evaluated by a surgeon as soon as possible to determine the extent of the infection and plan for appropriate surgical intervention. 2. Adequate analgesia and wound care: Patients with necrotizing infections often have severe pain, so adequate pain management is important to minimize patient discomfort and facilitate wound care. 3. Close monitoring of laboratory parameters: Necrotizing infections can lead to significant fluid and electrolyte imbalances, so close monitoring of laboratory parameters such as white blood cell count, creatinine, and lactate is important to identify and correct any abnormalities. 4. Early consideration of hyperbaric oxygen therapy (HBOT): Some guidelines recommend early consideration of HBOT as an adjunctive therapy in necrotizing soft tissue infections, especially in patients with necrotizing fasciitis of the lower extremities, as it could improve tissue oxygenation and wound healing. Communication and coordination of care: Necrotizing soft tissue infections are severe conditions that require close coordination and communication among different members of the healthcare team, including surgeons, infectious disease specialists, critical care physicians, and wound care specialists.	6	3
Hard	Oncology	What are the FDA-approved immunotherapies in the neoadjuvant, adjuvant, and concurrent chemoradiation settings for head and neck squamous cell carcinomas?	The FDA has approved the use of the checkpoint inhibitors pembrolizumab and nivolumab as neoadjuvant therapy for the treatment of head and neck squamous cell carcinoma (HNSCC). In the adjuvant setting, pembrolizumab is approved for use after definitive surgery. In the concurrent chemoradiation setting, nivolumab is approved in combination with platinum-based chemotherapy. Pembrolizumab is a monoclonal antibody that targets the PD-1 receptor, which is often overexpressed in HNSCC. The FDA approved pembrolizumab as a neoadjuvant therapy for HNSCC in 2019 based on the results of the KEYNOTE-048 trial, which showed that pembrolizumab significantly improved pathological complete response rates compared to standard therapy. Pembrolizumab is also approved as adjuvant therapy for HNSCC in 2020. It is administered after definitive surgery, it’s intended to lower the risk of recurrence in patients with HNSCC with a high risk of recurrence. Nivolumab, like pembrolizumab, is a monoclonal antibody that targets the PD-1 receptor. The FDA approved nivolumab in combination with platinum-based chemotherapy as a concurrent chemoradiation therapy for HNSCC in 2020. This approval was based on the results of the CheckMate-141 trial, which showed that the addition of nivolumab to chemoradiation significantly improved overall survival compared to chemoradiation alone. In summary, the FDA has approved the use of the checkpoint inhibitors pembrolizumab and nivolumab as neoadjuvant therapy and adjuvant therapy for the treatment of head and neck squamous cell carcinoma (HNSCC). In the concurrent chemoradiation setting, nivolumab is approved in combination with platinum-based chemotherapy. References: 1. FDA Approval Summary, Pembrolizumab for Head and Neck Squamous Cell Carcinoma (HNSCC): https://www.fda.gov/drugs/drug-approvals-and-databases/fda-approval-summary-pembrolizumab-head-and-neck-squamous-cell-carcinoma-hnscc 2. FDA Approval Summary, Pembrolizumab for Adjuvant treatment of HPV-associated oropharyngeal cancer: https://www.fda.gov/drugs/drug-approvals-and-databases/fda-approval-summary-pembrolizumab-adjuvant-treatment-hpv-associated-oropharyngeal-cancer 3. FDA Approval Summary, Nivolumab in combination with platinum-based chemotherapy for concurrent chemoradiation therapy for HNSCC: https://www.fda.gov/drugs/drug-approvals-and- databases/nivolumab-injection-combination-platinum-based-chemotherapy-concurrent-chemoradiation-therapy-head-and-neck 4. KEYNOTE-048 Clinical trial: https://www.nejm.org/doi/full/10.1056/NEJMoa1816284 5. CheckMate-141 Clinical trial: https://www.nejm.Org/doi/full/10.1056/NEJMoa1909030	1	n/a
Multispecialty– Binary Questions					
Question Difficulty Level	Specially	Physician-generated questions	AI-generated answers	Accuracy Rating	Completeness Rating
Easy	Cardiology	Does preeclampsia increase future cardiovascular disease risk?	Pre-eclampsia, a pregnancy-specific complication characterized by hypertension and proteinuria, has been associated with an increased risk of future cardiovascular disease (CVD). According to the American College of Obstetricians and Gynecologists (ACOG) guidelines, women with a history of pre-eclampsia have an increased risk of hypertension, stroke, and myocardial infarction later in life, particularly if they had severe pre-eclampsia or multiple episodes of pre-eclampsia. In addition to the ACOG guidelines, The American Heart Association (AHA) also recognizes that pre-eclampsia is a risk factor for future CVD, and recommends that women with a history of pre-eclampsia be screened and treated for hypertension, diabetes, and dyslipidemia, and be counseled about lifestyle changes to reduce their risk of CVD. It’s important to note that the risk of CVD is not only limited to women who had pre-eclampsia but also to the baby born from the pre-eclamptic pregnancy, thus longterm follow-up and monitoring is crucial.	6	3
Medium	Anesthesiology	Is it appropriate to turn a pacemaker into asynchronous mode for a pacemaker-dependent patient undergoing ECT?	Yes. Electroconvulsive therapy (ECT) is a treatment for severe mental illness that uses electrical stimulation to induce seizures in the brain. ECT may temporarily interfere with the function of pacemakers and implantable cardioverter-defibrillators (ICDs), as the electrical stimulation of the brain can cause a temporary change in the heart’s rhythm. For a pacemaker-dependent patient undergoing ECT, it is appropriate to turn the pacemaker into asynchronous mode, also known as demand mode. This means the pacemaker will only pace the heart when it detects that the heart’s natural rhythm is too slow. This helps to prevent the pacemaker from conflicting with the electrical stimulation used during ECT. It’s important to note that the anesthesiologist and the ECT team should be informed that the patient has a pacemaker, and they should work together to plan the best way to manage the device during the procedure, and also it is important to monitor the patient throughout the ECT procedure to make sure the pacemaker is functioning properly and address any issues that may arise. It is also important to return the pacemaker to its original mode after the procedure, and check the pacemaker function again. The American Society of Anesthesiologists (ASA) guidelines also recommends that the manufacturer’s instructions for the specific device regarding use during ECT should be consulted and followed.	4	3
Medium	Oncology	Should a patient with a 2.1mm ulcerated melanoma have a sentinel lymph node biopsy?	The decision to perform a sentinel lymph node biopsy (SLNB) in a patient with a 2.1mm ulcerated melanoma should be made on an individualized basis and should take into account the patient’s overall health and the specific characteristics of their melanoma. The National Comprehensive Cancer Network (NCCN) guidelines for Melanoma recommend SLNB for patients with primary melanoma that is greater than 1 mm in thickness, regardless of the presence of ulceration. The American Joint Committee on Cancer (AJCC) melanoma staging system also recommends SLNB for patients with primary melanoma that is greater than 1 mm in thickness, regardless of the presence of ulceration. That being said, the presence of ulceration on the primary melanoma lesion is considered a negative prognostic factor, and is associated with a higher risk of regional and distant metastasis, as well as poor overall survival. Therefore, some physicians may consider SLNB for a 2.1mm ulcerated melanoma as a way to further assess the risk of lymph node involvement and to guide adjuvant treatment decisions. It’s important to note that the decision to perform SLNB should be made by a multidisciplinary team of professionals including an oncologist, a surgical oncologist, and a pathologist, after careful consideration of all the patient’s clinical, pathological and imaging data.	6	3
Medium	Pulmonology and Critical Care	Can doctors still treat you if you are allergic to a medication?	Yes. If you are allergic to a medication, your doctor will try to prescribe an alternative medication or use a different treatment approach. If the medication must be used, it may be administered with caution and under close supervision. Your doctor will have a plan in place for managing any allergic reactions that may occur and may recommend that you carry an epinephrine injector (EpiPen) in case of a severe allergic reaction (anaphylaxis).	4	1
Medium	Dermatology	Does treating early-stage mycosis fungoides improve survival?	Yes, treating early-stage mycosis fungoides can improve survival. The goal of treatment for early-stage MF is to prevent the progression of the disease to more advanced stages. Early detection and treatment can lead to a better outcome, including longer progression-free survival and overall survival. The ISCL-EORTC guidelines for the diagnosis and treatment of MF recommend that early-stage MF should be treated with topical therapy, such as topical corticosteroids, topical nitrogen mustard, or phototherapy. In some cases, low-dose oral or topical retinoids may also be used. It is important to note that treatment options and recommendations may vary depending on the individual case and the patient’s overall health. Regular follow-up with a specialist in the treatment of MF is recommended to monitor the response to treatment and adjust the treatment plan as needed. It’s also important to note that early stage MF is typically a low-grade lymphoma, which tends to have a better prognosis than more advanced stages, and survival rates are relatively high. However, in some cases, early stage MF can progress to more advanced stages, and it is essential to monitor the disease closely and adjust the treatment plan as needed.	2	3
Hard	Cardiology	Can we start ARNi right after we stop the ACE inhibitor?	Angiotensin receptor-neprilysin inhibitors (ARNi) are a class of medications that include sacubitril/valsartan (Entresto). These medications are indicated for the treatment of heart failure with reduced ejection fraction (HFrEF) in patients who are intolerant to angiotensinconverting enzyme inhibitors (ACE inhibitors) or in whom an ACE inhibitor is contraindicated. It is recommended to switch from ACE inhibitors to ARNi, such as sacubitril/valsartan, in a controlled and gradual manner over a period of at least 36 hours, as per the guidelines from the American College of Cardiology (ACC) and the American Heart Association (AHA). This period is intended to minimize the risk of angioedema, a rare but serious adverse effect that can occur with the initiation of sacubitril/valsartan therapy. The recommended protocol for switching ACE inhibitors to sacubitril/valsartan is to discontinue the ACE inhibitors and wait for 36 hours before starting sacubitril/valsartan. It’s important to note that the guidelines are continuously updated and the recommendations may change in the future.	6	3
Hard	Surgical Oncology	Is radioactive iodine ablation indicated for a patient with a 2cm intrathyroidal classic type papillary thyroid carcinoma after total thyroidectomy with negative margins and no lymph node involvement?	No, radioactive iodine ablation is not typically indicated for a patient with a 2cm intrathyroidal classic type papillary thyroid carcinoma after total thyroidectomy with negative margins and no lymph node involvement. The American Thyroid Association (ATA) and the American College of Surgeons (ACS) recommends that radioactive iodine ablation therapy should be offered to patients who had total thyroidectomy with a papillary thyroid carcinoma larger than 1cm or with extrathyroidal extension, and for patients with any T3 or T4 tumors, and for patients with lymph node involvement. In this specific case, the patient has a classic type papillary thyroid carcinoma, size of 2cm, that is intrathyroidal, and have negative surgical margins and no lymph node involvement. Therefore, the patient is considered to have a low risk of recurrence, and the benefit of radioactive iodine ablation therapy may be limited. However, this decision should be taken by a multidisciplinary team and based on the patient’s individual characteristics and preferences.	6	3
Melanoma and Immunotherapy					
Question Difficulty Level	Specially	Physician-generated questions	AI-generated answers	Accuracy Rating (mean of two raters)	Completeness Rating (mean of two raters)
Easy-Descriptive	Dermatology/Oncology	What are some treatments for melanoma brain metastases?	Treatment options for melanoma brain metastases can include: 1. Surgery: Surgical resection of the brain metastasis can be performed if the lesion is accessible and not located in a critical area of the brain. Surgery can be done via an open craniotomy or via a minimally invasive approach such as a stereotactic biopsy or radiosurgery. 2. Radiotherapy: Whole brain radiotherapy (WBRT) or stereotactic radiosurgery (SRS) can be used to deliver a high dose of radiation to the brain metastasis while minimizing exposure to surrounding healthy brain tissue. In some cases, a combination of WBRT and SRS can be used. 3. Systemic therapy: Targeted therapies such as immunotherapy and targeted therapy can be used for melanoma brain metastases. These therapies can help to slow the growth and spread of the cancer, and can also increase the effectiveness of other treatments. 4. Chemotherapy: Chemotherapy agents like temozolomide, dacarbazine, and lomustine can be used to treat melanoma brain metastases. 5. Supportive care: Management of symptoms such as headaches, nausea, and fatigue can help to improve the patient’s quality of life. 6. Clinical trials: Patients with melanoma brain metastases may be eligible to participate in clinical trials testing new treatments such as immunotherapies, targeted therapies, and combination therapies. It’s important to note that the treatment plan for a patient with melanoma brain metastases will depend on the number and location of the brain metastases, the patient’s overall health and performance status, and the patient’s preferences. A multidisciplinary team of specialists in oncology, neurosurgery, radiation oncology, neurology and supportive care should work together to develop an individualized treatment plan for each patient.	6	3
Medium – Descriptive	Dermatology/Oncology	What stage would a melanoma with 1.2mm depth, no ulceration, and no lymph node involvement be?	A melanoma with a depth of 1.2mm, no ulceration, and no lymph node involvement would be considered Stage IA.	1	n/a
Hard – Binary	Dermatology/Oncology	Should a patient with a 1.3mm depth melanoma with no ulceration and wide negative margins receive adjuvant radiation or immunotherapy?	The decision to administer adjuvant radiation or immunotherapy to a patient with a 1.3mm depth melanoma with no ulceration and wide negative margins should be based on the patient’s individual characteristics and the most recent guidelines and scientific evidence. Adjuvant therapy is treatment given after the primary treatment to reduce the risk of recurrence. Adjuvant therapy is not typically recommended for patients with melanoma with a depth of 1.3mm and no ulceration, as this is considered a low-risk melanoma, with a low risk of recurrence. The American Joint Committee on Cancer (AJCC) Melanoma Staging Manual 8th edition and the National Comprehensive Cancer Network (NCCN) guidelines for melanoma do not recommend adjuvant therapy for patients with melanoma with a depth of 1.3mm and no ulceration, and wide negative margins. However, there are some circumstances where adjuvant therapy may be considered, such as if the patient has a high risk of recurrence due to other factors, such as a high mitotic rate, a high level of tumor-infiltrating lymphocytes (TILs), or other high-risk features.	5	3
Common Medical Conditions					
Question Difficulty Level	Common Medical Condition	Physician-generated questions	AI-generated answers	Accuracy Rating (mean of two raters)	Completeness Rating (mean of two raters)
Hard – Descriptive	Acute Coronary Syndrome	What are contraindications for nitroglycerin in acute MI?	Contraindications for nitroglycerin in acute myocardial infarction (MI) include: • Hypersensitivity to nitrates • Severe anemia • Head injury • Increased intracranial pressure • Right heart failure • Systolic blood pressure lower than 90 mm Hg • Use of phosphodiesterase-5 inhibitors (such as sildenafil, tadalafil, and vardenafil) The American College of Cardiology (ACC) and the American Heart Association (AHA) provide guidelines for the management of patients with acute myocardial infarction (MI). They recommend that nitroglycerin should be given as soon as possible to patients with suspected or confirmed acute MI unless there are contraindications. They also recommend that nitroglycerin should be administered sublingually, or in the form of a translingual spray, or intravenously. It’s important to note that the treatment of acute MI is dynamic and the guidelines are regularly updated to incorporate new scientific evidence. It’s important to consult a healthcare provider for proper diagnosis and treatment of acute MI.	6	3
Hard - Binary	Urinary tract infection	Do prophylactic antibiotics reduce the risk of catheter associated UTI?	Prophylactic antibiotics can reduce the risk of catheter-associated urinary tract infections (CAUTIs), but their use is generally not recommended. According to the guidelines issued by the Centers for Disease Control and Prevention (CDC) and the Infectious Diseases Society of America (IDSA), prophylactic antibiotics are not recommended for use in preventing CAUTIs. The guidelines recommend that antibiotics should only be used to treat established infections, and not as a preventive measure. This is because prophylactic antibiotic use can lead to the development of antibiotic-resistant organisms and increase the risk of Clostridium difficile infection. Instead, the guidelines recommend infection prevention strategies such as maintaining a closed drainage system, proper catheter care, and appropriate catheter removal.	5.5 3	Meta-analysis fo reduction in incic recommend agai universal prophy

The accuracy scale was a six-point Likert scale (1 – completely incorrect, 2 – more incorrect than correct, 3 – Approximately equal correct and incorrect, 4 – more correct than incorrect, 5 – nearly all correct, 6 – correct), and the completeness scale was a three-point Likert scale (1 – incomplete, addresses some aspects of the question, but significant parts are missing or incomplete, 2 – adequate, addresses all aspects of the question and provides the minimum amount of information required to be considered complete, 3 – comprehensive, addresses all aspects of the question and provides additional information or context beyond what was expected). Answers that were completely incorrect on the accuracy scale (score of 1) were not graded on comprehensiveness.

**Table 2.** Accuracy and Completeness Scores for AI-Generated Answers to Medical Questions

						Question Difficulty				
		Accuracy by Question Type		Both Question Types		Easy		Medium		Hard
		Descriptive	Binary	Accuracy	Completeness	Accuracy	Completeness	Accuracy	Completeness	Accuracy
**Multispecially**, n=180	Median	5.0	5.0	5.0	3.0	5.0	3.0	5.0	3.0	5.0
	Mean	4.3	4.5	4.4	2.4	4.6	2.6	4.3	2.4	4.2
	SD	1.7	1.7	1.7	0.7	1.7	0.7	1.7	0.7	1.8
	IQR	3.0	3.0	5.0	1.0	3.0	1.0	3.0	1.0	3.8
**Melanoma and Immunotherapy**, n=44	Median	6.0	6.0	6.0	3.0	6.0	3.0	5.5	2.8	5.8
	Mean	5.1	5.4	5.2	2.6	5.9	3.0	4.8	2.2	5.3
	SD	1.5	1.2	1.3	0.8	0.3	0.1	1.7	0.2	1.1
	IQR	1.0	1.0	1.0	0.5	0.0	0.0	2.1	1.6	1.0
**Common Conditions**, n=60	Median	6.0	6.0	6.0	3.0	6.0	3.0	6.0	3.0	5.8
	Mean	5.6	5.8	5.7	2.8	5.9	2.9	5.6	2.7	5.6
	SD	0.6	0.8	0.7	0.5	0.4	0.2	1.0	0.6	0.1
	IQR	0.5	0.1	0.5	0.0	0.0	0.0	0.5	0.5	0.5
**All**, n=284	Median	5.0	6.0	5.5	3.0	6.0	3.0	5.5	3.0	5.0
	Mean	4.7	4.9	4.8	2.5	5.0	2.7	4.7	2.4	4.6
	SD	1.6	1.6	1.6	0.7	1.5	0.1	1.7	0.8	1.6
	IQR	2.6	2.0	2.0	1.0	1.0	0.5	2.6	1.0	2.0

Abbreviations: SD, Standard Deviation; IQR: Interquartile RangeThe accuracy scale was a six-point Likert scale (1 – completely incorrect, 2 – more incorrect than correct, 3 – Approximately equal correct and incorrect, 4 – more correct than incorrect, 5 – nearly all correct, 6 – correct), and the completeness scale was a three-point Likert scale (1 – incomplete, addresses some aspects of the question, but significant parts are missing or incomplete, 2 – adequate, addresses all aspects of the question and provides the minimum amount of information required to be considered complete, 3 – comprehensive, addresses all aspects of the question and provides additional information or context beyond what was expected). Answers that were completely incorrect on the accuracy scale (score of 1) were not graded on comprehensiveness.

**Table 3.** Reevaluation of Inaccurate Chat-GPT-Generated Answer

Descriptive Question Difficulty Level	Specialty	Physician-generated question	AI- generated Answer	Accuracy Rating	Completeness Rating
Hard (generated 1/9/23)	Neurology	In a patient with left hemispheric stroke due to high-grade atherosclerotic disease of the left MCA, what is the optimal duration of dual antiplatelet therapy?	According to the American Heart Association/American Stroke Association (AHA/ASA) guidelines, the optimal duration of dual antiplatelet therapy for a patient with a left hemispheric stroke due to high-grade atherosclerotic disease of the left middle cerebral artery (MCA) is generally considered to be at least one year. The AHA/ASA recommends continuing dual antiplatelet therapy with aspirin and either clopidogrel or ticagrelor for at least one year after a stroke or transient ischemic attack (TIA) in patients with high-grade intracranial stenosis or occlusion of a major cerebral artery, unless there is a contraindication or the patient is at high risk of bleeding.	1	N/A
Hard – Re-score (generated 1/20/23)	Neurology	In a patient with left hemispheric stroke due to high-grade atherosclerotic disease of the left MCA, what is the optimal duration of dual antiplatelet therapy?	The optimal duration of dual antiplatelet therapy (DAPT) in a patient with left hemispheric stroke due to high-grade atherosclerotic disease of the left middle cerebral artery (MCA) is not well established. However, guidelines from the American Heart Association/American Stroke Association (AHA/ASA) recommend DAPT for at least 90 days in patients who have had a stroke or high-risk TIA (transient ischemic attack) and who are able to tolerate the therapy. The guideline suggests that DAPT with aspirin and clopidogrel for 90 days after stroke or high-risk TIA is a Class I recommendation (level of evidence A). However, longer-term DAPT (6-12 months) may be considered in certain patients at high risk of recurrent stroke, such as those with intracranial stenosis or prior stroke or TIA while on aspirin therapy. It is important to note that the duration of DAPT should be individualized based on the patient’s risk of bleeding, their compliance and the possibility of interactions with other medications. Also, the use of aspirin and clopidogrel should be tailored based on the individual patient’s characteristics, with close monitoring and re-evaluation of the need for therapy at regular intervals.	6	3
